# P-685. RSV Viral Shedding Over the Time Course of Infection: PCR Positivity and Time to Last Positive Test

**DOI:** 10.1093/ofid/ofaf695.898

**Published:** 2026-01-11

**Authors:** J Bradford Bertumen, Sara Benist, Sarah E Smith-Jeffcoat, E Ivy Oyegun, Erin South, Jonathan Schmitz, Yuwei Zhu, Keipp Talbot, Carlos G Grijalva, Son H McLaren, Ellen Sano, Celibell Vargas, Melissa Stockwell, Natalie K Lo, Sandra McAteer, Erica Clark, Helen Y Chu, Hannah L Kirking

**Affiliations:** Centers for Disease Control and Prevention, DECATUR, GA; Centers For Disease Control and Prevention, Atlanta, Georgia; Centers for Disease Control and Prevention, DECATUR, GA; CDC, Atlanta, Georgia; Centers for Disease Control and Prevention, DECATUR, GA; Vanderbilt University Medical Center, Nashville, TN; VUMC, NASHVILLE, Tennessee; Vanderbilt University Medical Center, Nashville, TN; Vanderbilt University Medical Center, Nashville, TN; Columbia University Irving Medical Center, New York City, New York; Columbia University Irving Medical Center, New York City, New York; Columbia University Irving Medical Center, New York City, New York; Columbia University Irving Medical Center, New York City, New York; University of Washington, Seattle, Washington; University of Washington, Seattle, Washington; University of Washington, Seattle, Washington; University of Washington, Seattle, Washington; Coronavirus and Other Respiratory Viruses Division, National Center for Immunization and Respiratory Diseases, CDC, Atlanta, GA

## Abstract

**Background:**

Community-based data on respiratory syncytial virus (RSV) shedding are limited but crucial to understanding viral transmission. This analysis assesses polymerase chain reaction (PCR) positivity trajectories among ambulatory people with RSV infection.

**Figure 1:**
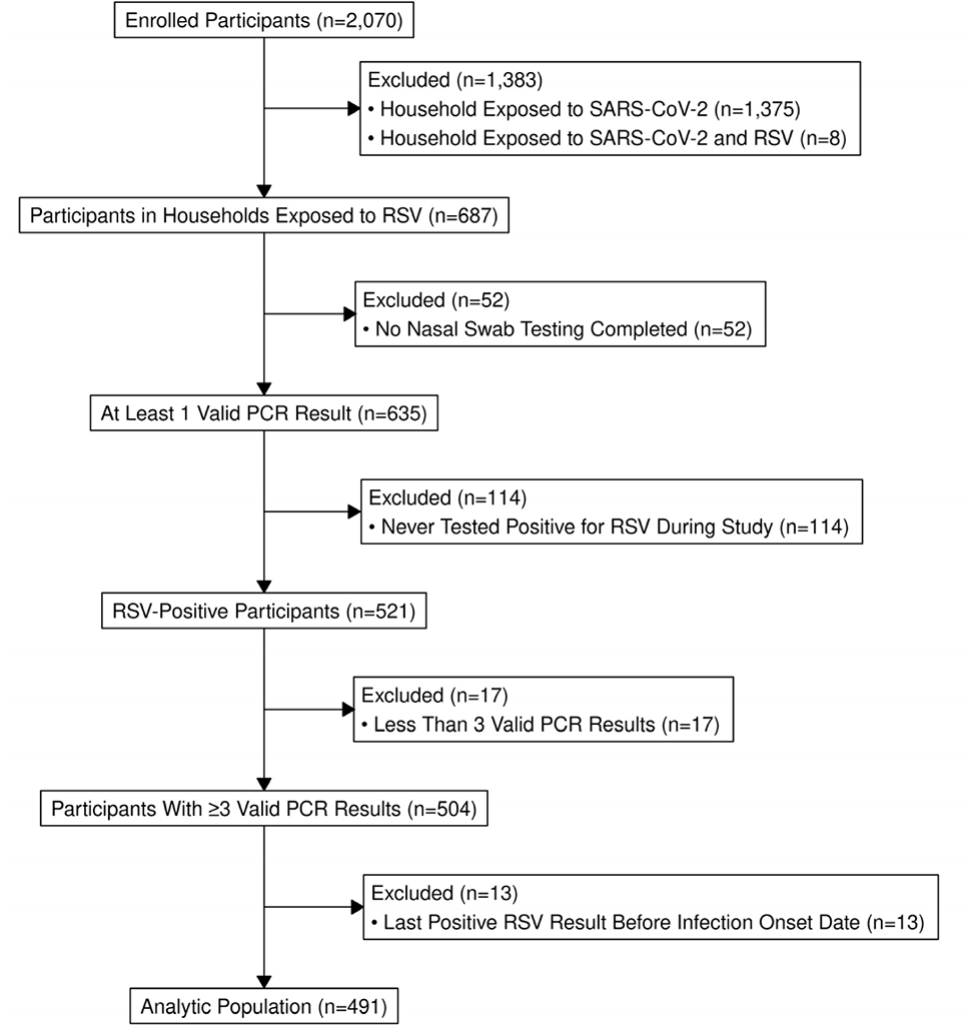
Participants enrolled in a case-ascertained household transmission study and included in analysis, Tennessee, New York, and Washington, January 2024 ─ January 2025 Participants were excluded from the analysis if they came from the household of an index case who initially tested positive for SARS-CoV-2 with or without testing positive for RSV, never tested positive for RSV, did not have at least 3 valid PCR results, or their last known positive PCR test occurred before their infection onset dates and therefore positivity since day of onset could not be determined.

**Table 1:**
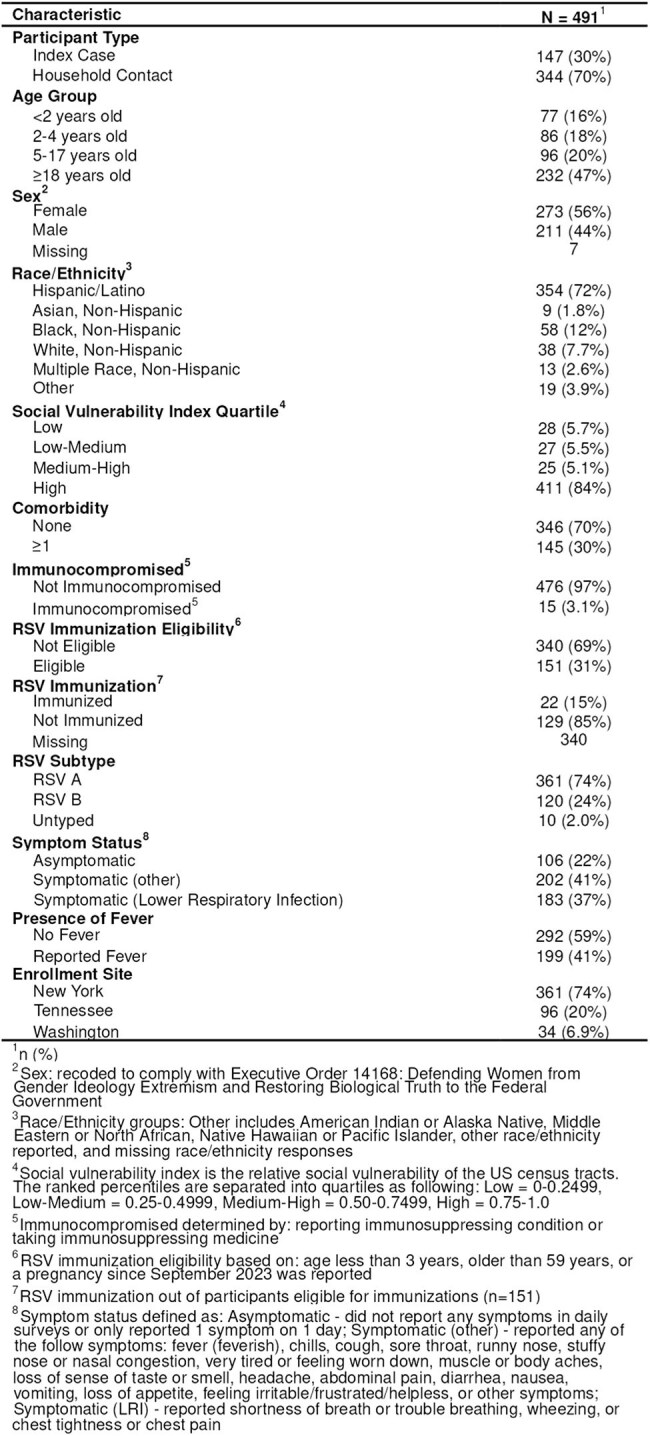
Demographic, clinical, and epidemiologic characteristics of participants with RSV infection, January 2024 ─ January 2025

**Methods:**

Included participants were enrolled in an RSV household transmission study from 1/2024 ─ 1/2025, tested positive for RSV, and had ≥ 3 valid PCR results. Participants were followed for 10 days, during which daily symptoms and self-collected nasal swabs were obtained. Swabs were tested using Hologic Panther® PCR assay. Infection onset was defined as the first day of symptoms. For participants who were asymptomatic, onset date was assigned by adding an imputed serial interval to the onset date of the initial household case, all of whom were symptomatic. Percent positivity (95% confidence intervals (CI)) was calculated as number of positive PCR tests over total valid test results on each day since onset. Kaplan-Meier survival analyses and log-rank tests were used to compare median days from onset to last positive PCR test by age and symptoms.

**Figure 2:**
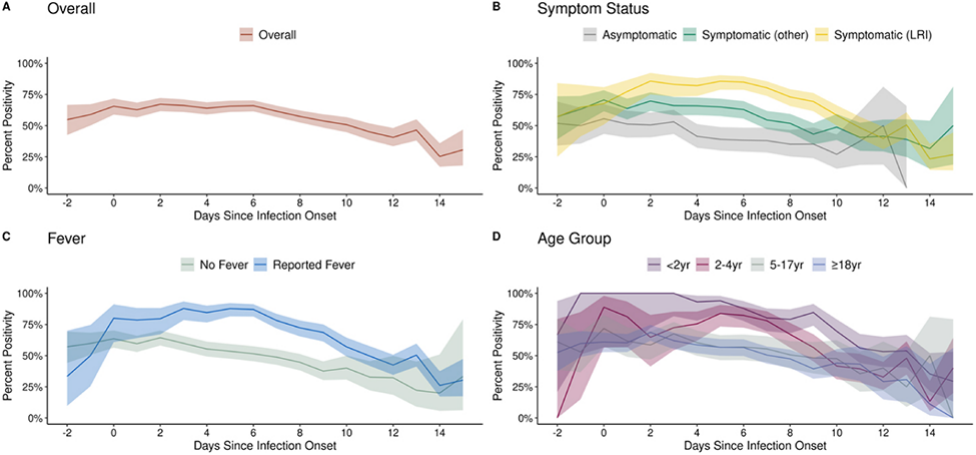
Respiratory syncytial virus (RSV) percent positivity by day since infection onset overall (A), by symptom status (B), by fever status (C), and by age group (D)

The dark lines represent the daily RSV percent positivity of all specimens tested on each day since onset with shaded areas representing the Wilson binomial 95% confidence intervals. Participants completed retrospective daily symptom surveys for the time between index onset and enrollment (up to 6 days) and prospective daily symptom surveys starting at enrollment for 10 days with concurrent nasal swab collection. Infection onset was defined as the first day of symptoms. If the participant was asymptomatic, onset date was assigned by adding an imputed serial interval to the onset date of the initial household case, all of whom were symptomatic. This serial interval was imputed based on symptomatic participants with similar characteristics as the asymptomatic participant. Lower respiratory infection (LRI) symptoms included shortness of breath/trouble breathing, wheezing, or chest tightness/chest pain. Other symptoms included fever, chills, cough, sore throat, runny nose, stuffy nose/nasal congestion, very tired/feeling worn down, muscle/body aches, loss of sense of taste or smell, headache, abdominal pain, diarrhea, nausea, vomiting, loss of appetite, feeling irritable/frustrated/helpless, or other symptom. Asymptomatic was defined as never reporting any symptom or only ever reporting one symptom on one day. Fever included measured fever and subjective fever (i.e., feeling feverish).

**Figure 3:**
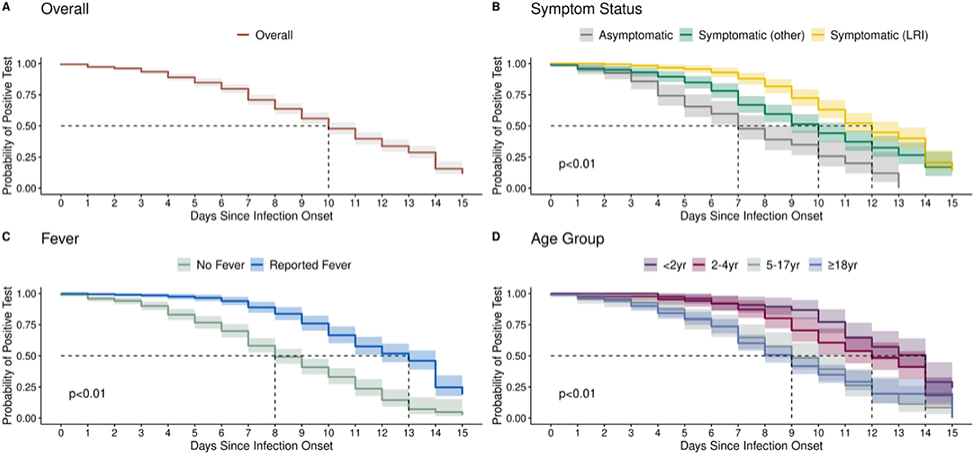
Time to last respiratory syncytial virus (RSV) positive PCR test result using Kaplan-Meier survival analysis overall (A), by symptom status (B), by fever status (C), and by age group (D).

Kaplan-Meier survival analysis was used to determine the time from infection onset to last positive PCR test. The shaded areas represent 95% confidence intervals, the dotted lines represent the median time to last positive test. The log-rank test was performed to compare the curves between groups. Participants completed retrospective daily symptom surveys for the time between index onset and enrollment (up to 6 days) and prospective daily symptom surveys starting at enrollment for 10 days with concurrent nasal swab collection. Infection onset was defined as the first day of symptoms. If the participant was asymptomatic, onset date was assigned by adding an imputed serial interval to the onset date of the initial household case, all of whom were symptomatic. This serial interval was imputed based on symptomatic participants with similar characteristics as the asymptomatic participant. Lower respiratory infection (LRI) symptoms included shortness of breath/trouble breathing, wheezing, or chest tightness/chest pain. Other symptoms included fever, chills, cough, sore throat, runny nose, stuffy nose/nasal congestion, very tired/feeling worn down, muscle/body aches, loss of sense of taste or smell, headache, abdominal pain, diarrhea, nausea, vomiting, loss of appetite, feeling irritable/frustrated/helpless, or other symptom. Asymptomatic was defined as never reporting any symptom or only ever reporting one symptom on one day. Fever included measured fever and subjective fever (i.e., feeling feverish).

**Results:**

A total of 491 participants were included (Figure 1); 273 (56%) were female, 163 (34%) were < 5 years old, and 385 (78%) reported symptoms with 199 (41%) reporting fever (Table 1). Overall peak percent positivity was 67% (CI: 62%, 72%) at day 2 after onset; 88% (CI: 78%, 94%) on day 3 for those with fever; 86% (CI: 75%, 92%) on day 2 for those with lower respiratory infection (LRI) symptoms; 100% (CI: 51%, 100%) on day -1 (day before onset) for those < 2 years old; and 89% (CI: 57%, 98%) on day 0 for those 2-4 years old (Figure 2). Overall median time to last positive test was 10 days (CI: 10,11). Median time to last positive test was longer for those with fever vs without fever (13 vs 8 days; log-rank p< 0.01) and for those with LRI symptoms vs without LRI symptoms (12 vs 9 days; log-rank p< 0.01) (Figure 3). Median time to last positive test was longest for the youngest participants (14 days for < 2 years old, 12 days for 2-4 years old, and 9 days for ≥ 5 years old; log-rank p< 0.01).

**Conclusion:**

Fever, LRI symptoms, and age < 5 years were associated with higher RSV positivity and prolonged viral shedding in persons with RSV. Persons with these characteristics may be at higher risk of transmitting RSV in the community.

**Disclosures:**

Carlos G. Grijalva, MD MPH, AHRQ: Grant/Research Support|CDC: Grant/Research Support|GSK: Advisor/Consultant|Merck: Advisor/Consultant|NIH: Grant/Research Support|Syneos Health: Grant/Research Support Melissa Stockwell, MD MPH, CDC: Grant/Research Support Helen Y. Chu, MD, MPH, Roche: Advisor/Consultant|Vir: Advisor/Consultant

